# Impact of Phytomediated Zinc Oxide Nanoparticles on Growth and Oxidative Stress Response of *In Vitro* Raised Shoots of *Ochradenus arabicus*

**DOI:** 10.1155/2021/6829806

**Published:** 2021-12-06

**Authors:** Fahad Al-Qurainy, Salim Khan, Saleh Alansi, Mohammad Nadeem, Aref Alshameri, Abdel-Rhman Gaafar, Mohamed Tarroum, Hassan O. Shaikhaldein, Abdalrhaman M. Salih, Norah Arrak Alenezi, Norah S. Alfarraj

**Affiliations:** Department of Botany and Microbiology, College of Science, King Saud University, Riyadh 11451, Saudi Arabia

## Abstract

Biogenic nanoparticles have potential roles in the growth and development of plants and animals as they are ecofriendly and free of chemical contaminants. In this study, we assessed the effects of phytomediated zinc oxide nanoparticles (ZnONPs) on shoot growth, biochemical markers, and antioxidant system response in *Ochradenus arabicus*, which is a medicinal plant. The shoot length and fresh and dry weights were found to be higher in groups with 5 and 10 mg/L ZnONPs than in the control. At high concentrations of ZnONPs (50, 100, and 300 mg/L), biomass was decreased in a concentration-dependent manner. The shoot number was observed to be highest at 50 mg/L among all applied concentrations of ZnONPs. The levels of the stress markers proline and TBARS were found to be higher in shoots treated with 100 and 300 mg/L ZnONPs than in the control as well as NP-treated shoots. The levels of antioxidant enzymes were significantly increased at high concentrations of nanoparticles compared with the control. Thus, synthesized phytomediated ZnONPs from shoots of *O. arabicus* and their application to the same organ of *O. arabicus in vitro* were found to be effective as a low concentration of nanoparticles promoted shoot growth, resulting in high biomass accumulation. Thus, using green nanotechnology, such endemic plants could be conserved *in vitro* and multiple shoots could be produced by reducing the phytohormone concentration for multiple uses, such as the production of potential secondary metabolites.

## 1. Introduction

The application of nanoparticles (NPs) in agriculture has contributed to increased crop quality, the regulation of crop production, and improved stress tolerance in various plant species. At present, various methods are available for synthesizing NPs, such as chemical and physical methods. However, they can be expensive, environmentally damaging, and labor-intensive. Nanoparticles have both benefits and risks for flora and fauna. Substantial work has been carried out on the synthesis of nanoparticles and their use in biological systems [1, 2]. However, work related to nanoparticle applications on plant systems has been limited to the observation of physiological, biochemical, and morphological parameters. The phytomediated synthesis of NPs is nontoxic, inexpensive, facile, ecofriendly, and beneficial not only to human health but also to different types of microorganisms. Zinc nanoparticles (used as micronutrients in agriculture) have potential to boost the yield and growth of medicinal as well as food crops. Zinc oxide nanoparticles (ZnONPs) are among the most commonly synthesized NPs worldwide, next to carbon nanotubes and gold, silver, and titanium dioxide NPs [3]. In the last few years, ZnONPs have received significant attention due to their purported ability to enhance nutrient accumulation by plants in order to augment the quality of crops [4, 5]. Some nanoparticles have been used for various biological purposes, such as gene and drug delivery. Various metallic oxide nanoparticles have been used for growth promotion as well as secondary metabolite production from candy leaf [6, 7].

Zinc is considered an important micronutrient for both animals and plants [8]. It maintains the integrity of the cell membrane, ensures chloroplast synthesis, and enhances enzyme activities. According to Cakmak [9], Zn exerts effects on membrane function, protein synthesis, cell elongation, and environmental stress tolerance. Zinc is a necessary micronutrient and plays potential roles in the activities of various enzymes, such as tryptophan synthetase, dehydrogenases, transphosphorylases, aldolases, superoxide dismutase, isomerases, and DNA and RNA polymerases [10, 11]. Farmers use zinc fertilizers to enhance crop yield. Zinc can also control some diseases caused by bacteria and fungi. Moreover, Zn plays important roles in the regulation of phytohormones such as auxin, protein synthesis, carbohydrate metabolism, and stress-alleviating responses [12, 13]. Various nanoparticles synthesized by plant extracts have many promoting effects on seed germination, morphological markers, yield traits, and biomass [14].

*Ochradenus arabicus* (family: Resedaceae) is a medicinal plant used locally and found mostly in desert regions of Saudi Arabia. Different compounds have been reported from the genus *Ochradenus*, such as quercetin-3-O-p-coumaryl(1-6)-*β*-glucosyl(1-6)-*β*-glucoside-7-O-*α*-rhamnoside, quercetin-3-O-*β*-glucosyl(1-2)-*α*-rhamnoside-7-O-*α*-rhamnoside, quercetin glycosides, isoquercitrin, quercetin-3-gentiobioside, and others including astragalin, kaempferol, glycosides, and afzelin [15]. Various solvent extracts of *O. arabicus* were reported to show antibacterial, anticancer, and antifungal activities [16]. The plant has seed dormancy, which may be due to the hard seed coat, presence of some inhibitors, underdeveloped embryos, or low internal hormone levels. The germination rate of *O. arabicus* is very low at 2.6%, as reported by Nadeem et al. [17].

A high concentration of ZnONPs produces oxidative stress, which is common in plants; however, in response to NPs, plants produce antioxidant enzymes and secondary metabolites to cope with oxidative stress. There are a few reports on the application of NPs for *in vitro* plant growth and development. However, there is a need to investigate the effects of such NPs on plant growth and plant regeneration as plants have potential applications to cure various health problems due to the presence of different secondary metabolites. Unfortunately, *O. arabicus* is endemic to Saudi Arabia and found in only a few places within the country [18], so our aim is to produce multiple *O. arabicus* shoots with high biomass by applying phytomediated ZnONPs. Therefore, in the present study, we investigated the effect of phytomediated ZnONPs synthesized from *O. arabicus* and their application to regenerated shoots of this species for the first time.

## 2. Materials and Methods

### 2.1. Seed Germination

Seeds of wild plant *Ochradenus arabicus* were collected from the Central Region of Saudi Arabia in 2010, under the project (Grant No: 10-BIO1289-02) approved by the National Plan for Science, Technology, and Innovation. The plant was identified by a taxonomist, Dr. Jacob Thomas Pandalayil, at the Department of Botany and Microbiology, College of Science, King Saud University, and identification was also performed by internal transcribed spacer sequence of ribosomal DNA (GenBank accession No: JQ899053). Mature seeds were washed with tap water for 30 min to remove dust and microbes. The washed seeds were kept in 50% bleach for 20 min for surface disinfection, after which they were washed with autoclaved distilled water three or four times. Seeds were kept for germination on 0.8% agar in the dark at 25 ± 1°C.

### 2.2. Shoot Proliferation from Germinated Seeds

Shoots were produced on MS medium [19] with cytokinin (1 mg/L BA). The cultures were grown in a culture room set at relative humidity 55%–60% and temperature 25°C–28°C under PPFD of 40 *μ*mol m^−2^ s^−1^ for a 16 h photoperiod. The shoots were cultured for 45 days on MS medium to obtain more shoots.

#### 2.2.1. Plant Extract Preparation for ZnONP Synthesis

Young shoots of *O. arabicus* obtained *in vitro* on MS medium were used for nanoparticle synthesis. Five grams of fresh shoots was chopped with a razor into small pieces to obtain a good amount of extract. To prepare the shoot extract, these small pieces were added to 100 mL of double-distilled water (in a 250 mL conical flask) and kept in a water bath at 100°C for 20 min. The extract was taken out from the water bath and cooled down. This extract was then filtered with Whatman filter paper No. 1 twice and kept at 4°C until use.

#### 2.2.2. Phytomediated Synthesis of ZnONPs

A total of 100 mL of fresh *O. arabicus* shoot extract was added to 0.05 M Zn(NO_3_)_2_ solution at 60°C, followed by continuous stirring for 12 h. A brown whitish color appeared upon the addition of 0.1 N NaOH, as upon the synthesis of nanoparticles. Centrifugation was performed at 8000 rpm, 4°C for 10 min for pellet formation. The pellet was washed with double-distilled water. Further, the pellet was washed with absolute alcohol and dried at 40°C for 12 h. Calcination was performed at 500°C for 3 h.

### 2.3. Characterization of ZnONPs

The biologically synthesized ZnONPs were observed as precipitation of brown whitish particulates and confirmed by UV-visible spectroscopy. Particle shape, size, and texture were analyzed using Fourier transform infrared (FTIR) and transmission electron microscopy (TEM). Dynamic light scattering (DLS) was also used to measure the size of the synthesized nanoparticles.

#### 2.3.1. Optimization of ZnONP Concentration

The stability of the synthesized ZnONPs was evaluated using UV-Vis spectroscopy before their application on shoots. Various concentrations of ZnONPs, including 5, 10, 25, 50, 100, and 300 mg/L, were applied to the regenerated shoots of *O. arabicus* cultured on MS medium to determine the optimum growth and shoot proliferation with phytohormone (0.1 mg/L BA). As a control, the culture without nanoparticles but the same concentration of BA (0.1 mg/L BA) was performed. Shoot explants of the same size for the control as well as the treatments were kept on MS medium. Each magenta jar contained 100 mL of MS medium, and four explants were kept to determine the reproducibility of the results. The cultures were grown in a culture room set at relative humidity 55%–60% and temperature 25°C–28°C under PPFD of 40 *μ*mol m^−2^ s^−1^ for a 16 h photoperiod. The shoots were cultured for 45 days on MS medium to investigate growth parameters.

#### 2.3.2. Biomass Determination

The fresh and dry weights of treated and untreated shoots were measured after 45 days of culture on MS medium. Each treatment was performed in triplicate to ensure reliability of the results.

#### 2.3.3. Shoot Length and Number

Shoot length and number were measured after 45 days of culture on MS medium and were compared with the control and among the treated shoots.

#### 2.3.4. Estimation of Total Chlorophyll

The method reported by Arnon [20] for estimating total chlorophyll was used. Fresh leaves (0.1 g) were washed in DW, chopped into small pieces, and extracted in DMSO. Incubation was performed at 65°C for 120 min, after which the samples were taken out and absorbance was measured immediately at 663 nm and 645 nm using a UV-Vis spectrophotometer. The calculated chlorophyll content was expressed as mg/g FW.

#### 2.3.5. Proline Estimation

Fresh leaves (0.5 g) were used for the estimation of proline using the method developed by Hanson et al. [21]. The fresh samples were ground in 10 mL of 3% aqueous sulfosalicylic acid. The samples were centrifuged for 15 min at 9000 × g, and the obtained supernatant (2 mL) was taken out and placed in another tube. Two milliliters of each acid ninhydrin and acetic acid was added to the above samples. The samples were incubated in boiling water for 1 h, and the reaction was terminated by putting the samples in an ice bath. Toluene (4 mL) was added to the above samples and vortexed. The aqueous phase was separated from chromatophore-containing toluene. The proline content was estimated in samples by measuring the absorbance of chromatophore-containing toluene at 520 nm (model UB-1800; Shimadzu, Japan).

#### 2.3.6. Thiobarbituric Acid Reactive Substances (TBARS)

TBARS content of fresh leaves was estimated using the method of Cakmak and Horst [22]. The leaf samples (0.5 g) were homogenized in 5 mL of trichloroacetic acid (TCA, 0.1% (*w*/*v*)). The supernatant was collected at 12,000 × g for TBARS estimation. Four milliliters of 0.5% (*w*/*v*) thiobarbituric acid (TBA) in 20% (*w*/*v*) TCA was added to 1 mL of supernatant taken from the above step and placed for 30 min at 90°C in a water bath. The reaction was stopped by placing the sample in an ice bath, followed by centrifugation at 10,000 × g, after which the supernatant was collected. TBARS content was measured from the absorbance measured at wavelengths of 532 and 600 nm with a spectrophotometer:

(1) $$\begin{array}{c} \text{TBARS }\left(\text{nmol }{\text{g}}^{-1}\text{ FW}\right)=\frac{\text{A}532-\text{A}600\times V\times 1000}{155\left(\text{extinction coefficient}\right)\times W}. \end{array}$$

Here, A532 represents absorbance at 532 nm, A600 represents absorbance at 600 nm, *V* is extraction volume, and *W* is fresh weight of tissue.

#### 2.3.7. Catalase (EC 1.11.1.6)

The method developed by Aebi [23] was used to record the activity of catalase (CAT). A total of 0.25 g of fresh leaves was ground in phosphate buffer (0.5 M, pH 7.3) containing 0.3 mM EDTA, 1% Triton ×100 (w/v), and 1% PVP (*w*/*v*). The mixture was centrifuged, and the supernatant was collected for an enzymatic assay. The enzyme activity assay was performed in 2 mL of reaction buffer containing 0.1 mL of 3 mM EDTA, 0.1 mL of enzyme extract, and 0.1 mL of 3 mM H_2_O_2_ for 3 min. CAT was recorded at 240 nm using a UV-Vis spectrophotometer, with enzyme unit expressed in mg^−1^ protein min^−1^.

#### 2.3.8. Superoxide Dismutase (EC 1.15.1.1)

The superoxide dismutase (SOD) activity was measured using the method reported by Dhindsa et al. [24]. The fresh leaf samples (0.25 g) were ground in 2 mL of phosphate buffer containing 1% Triton ×100 (*w*/*v*), 0.3 mM EDTA, and 1% PVP (*w*/*v*). The sample was centrifuged at 10,000 × g for 10 min, after which the supernatant was collected for assaying SOD activity. The enzyme assay was performed in 1.5 mL of reaction buffer containing 0.2 mL of methionine and 0.1 mL of each of 1 M NaCO_3_, 2.25 mM NBT solution, riboflavin, 3 mM EDTA, enzyme extract, and 1 mL of DDW incubated in the light. The blank was kept in the dark while containing all components as in the treated samples. The sample absorbance along with the blank was recorded at 560 nm using a UV-Vis spectrophotometer (Model UB-1800; Shimadzu, Japan). A 50% reduction in color was considered one enzyme unit (EU), and activity of SOD was calculated in mg^−1^ protein min^−1^.

#### 2.3.9. Ascorbate Peroxidase (EC 1.11.1.11)

The ascorbate peroxidase (APX) activity was checked using the method of Nakano and Asada [25]. Fresh leaves (0.25 g) were ground in 1 mL of extraction buffer (50 mM phosphate buffer), 1% Triton ×100 (*w*/*v*), 1% PVP (*w*/*v*), and 0.3 mM EDTA. The supernatant was obtained after centrifugation for assaying enzyme activity at 290 nm using a UV-Vis spectrophotometer. The extinction coefficient (*e*) 2.8 mM^−1^ cm^−1^ was used for calculating APX activity and expressed as EU mg^−1^ protein min^−1^.

#### 2.3.10. Glutathione Reductase (EC 1.6.4.2)

The glutathione reductase (GR) activity was measured in fresh shoots at a wavelength of 340 nm following the protocol of Rao [26]. The fresh leaves (0.25 g) were ground in extraction buffer, and the supernatant was collected after centrifugation for the enzymatic assay. The reaction mixture (1 mL) contained 0.05 mL of each of 0.2 mM NADPH and 0.5 mM GSSG and 0.1 mL of enzyme extract. The GR activity was calculated using the molar absorptivity constant of NADPH (6.2 mM^−1^ cm^−1^) and expressed as EU mg^−1^ protein min^−1^.

### 2.4. Statistical Analysis

The experimental design was based on triplicates, repeated twice, and was conducted under controlled conditions. ANOVA (post hoc Duncan) was used to test the experimental data using Origin Pro software (v.8.5). All values are presented as the mean ± SD.

## 3. Results

### 3.1. Characterization of Phytomediated ZnONPs

**Figure 1:**
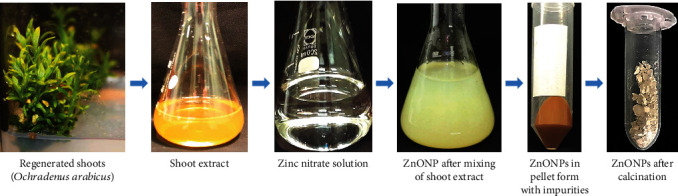
Phytomediated ZnONP synthesis from regenerated shoots of *Ochradenus arabicus.*

**Figure 2:**
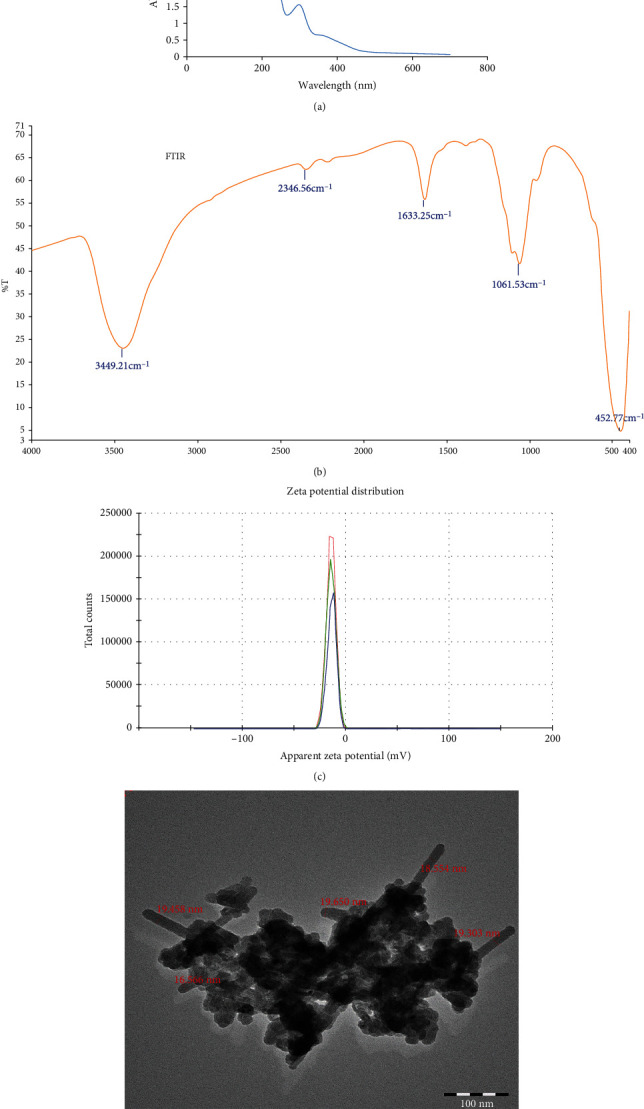
Characterization of synthesized ZnONPs with various techniques. (a) UV spectra of ZnONP; (b) Fourier transform infrared (FTIR) spectra; (c) zeta potential; (d) TEM micrograph of ZnONPs.

Fresh shoots with leaves (45 days old) raised on MS medium [19] were used for the preparation of ZnONPs, as shown in Figure 1. The optical absorption spectrum of the synthesized ZnONPs was recorded in the range of 200–800 nm using a UV-Vis spectrophotometer (Shimadzu). The UV spectrum was taken at different time intervals to evaluate the synthesis of nanoparticles. The white precipitation in the suspension indicated the synthesis of ZnONPs, and a further sharp peak of the UV spectrum at 280 nm showed the pure synthesis of ZnONPs (Figure 2(a)). The sharp peak indicated the synthesis of nanoparticles with a nanoscale size and distribution [27]. The powder of ZnONPs was used for obtaining Fourier transform infrared (FTIR) spectra (as pellets in KBr) in the range of 4000–400 cm^−1^ with a resolution of 1 cm^−1^ (Figure 2(b)). The FTIR results revealed the presence of functional groups in the shoot extract, which are responsible for the reduction and stabilization of zinc oxide nanoparticles. FTIR spectra obtained for ZnONPs showed shifts and a number of peaks, which indicated their complex nature.

An absorption band was identified as a broad and intense peak at 3449.21 cm^−1^, resulting from stretching and vibration mode of O-H bond [28]. The peak at 2346.56 cm^−1^ corresponded to CO_2_ that was adsorbed on the surface of the sample [29]. The peak at 1633.25 cm^−1^ was assigned to a *β*-sheet, as a secondary structure of the protein [30]. The peak at 1061.53 cm^−1^ was attributed to stretching of the carbonyl functional groups in carboxylic acids, ketones, and aldehydes [31]. The band at 452.77 cm^−1^ was assigned to the stretching vibration mode of the Zn-O bond in ZnO nanoparticles. These results confirmed the presence of functional groups in the shoot extract of *O. arabicus* which are responsible for reduction and stabilization of synthesized zinc oxide nanoparticles.

The average zeta potential value of ZnONPs was observed to be −14.5 mV with conductivity of 0.0148 mS/cm (Figure 2(c)), which indicated their stability. From the TEM micrograph of ZnONPs, they were observed to be in the size range of 16.56–19.65 nm (Figure 2(d)).

### 3.2. Measurement of Morphometric Traits and Biomass

**Figure 3:**
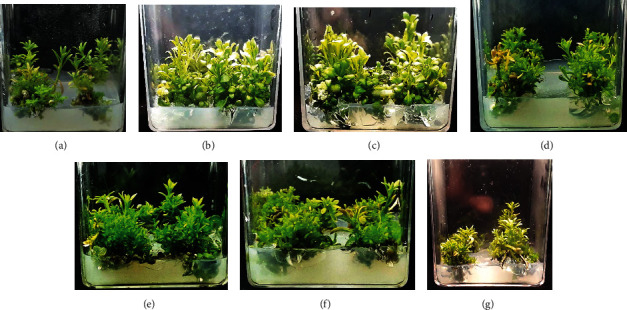
Shoot growth of *Ochradenus arabicus* on MS medium with different concentrations of ZnONPs: (a) control; (b) shoot growth with 5 mg/L; (c) shoot growth with 10 mg/L; (d) shoot growth with 25 mg/L; (e) shoot growth with 50 mg/L; (f) shoot growth with 100 mg/L; (g) shoot growth with 300 mg/L.

**Figure 4:**
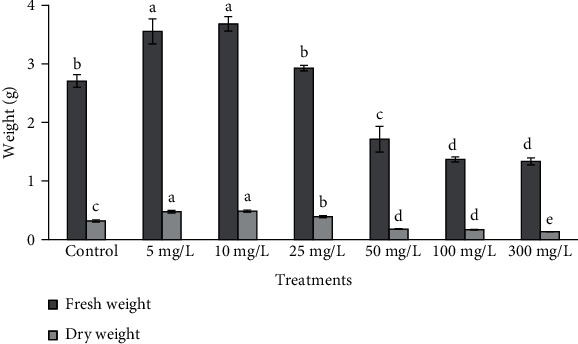
Fresh and dry weight of *Ochradenus arabicus* shoots grown at different concentrations of ZnONPs. Various letters on bars show the significant values according to Duncan's test (*p* < 0.05).

**Figure 5:**
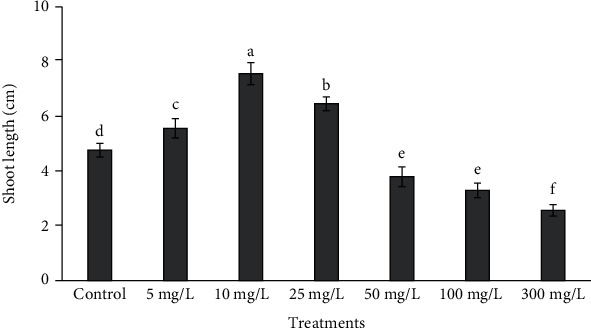
Shoot length variation at different concentrations of ZnONPs. Various letters on bars show the significant values according to Duncan's test (*p* < 0.05).

**Figure 6:**
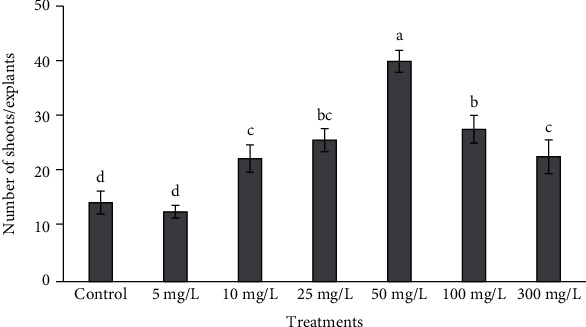
Shoot number observed on MS medium at different concentrations of ZnONPs. Various letters on bars show the significant values according to Duncan's test (*p* < 0.05).

Morphometric traits including shoot length and number varied at different concentrations of ZnONPs, and some of them were found to be better than control as well as other shoot treatments (Figure 3). The highest biomass at 5 and 10 mg/L ZnONPs and shoot length at 10 and 25 mg/L ZnONPs were observed upon comparison with the control as well as other NP-treated shoots (Figures 4 and 5). As the concentrations of phytomediated ZnONPs increased in MS medium (50, 100, and 300 mg/L), the shoot length was affected more than the shoots treated with low concentrations of ZnONPs (5 and 10 mg/L) and the control shoots. The shoot number was higher significantly than the control with ZnONP concentrations 10, 25, 50, 100, and 300 mg/L as compared to control. Among all treatments, the shoot number was found to be highest at 50 mg/L ZnONPs (Figure 6).

### 3.3. Biochemical Parameters

**Figure 7:**
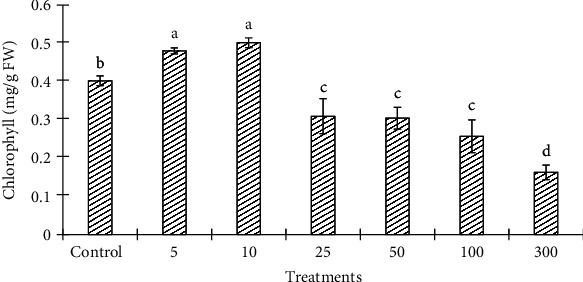
Chlorophyll content in the leaves of *Ochradenus arabicus* treated with different concentrations of ZnONPs. Various letters on bars show the significant values according to Duncan's test (*p* < 0.05).

**Figure 8:**
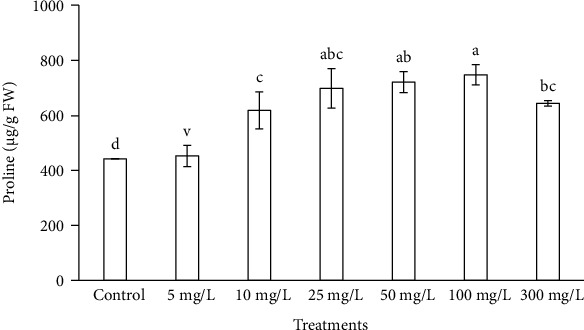
Proline content in the leaves of *Ochradenus arabicus* treated with various concentrations of ZnONPs. Various letters on bars show the significant values according to Duncan's test (*p* < 0.05).

**Figure 9:**
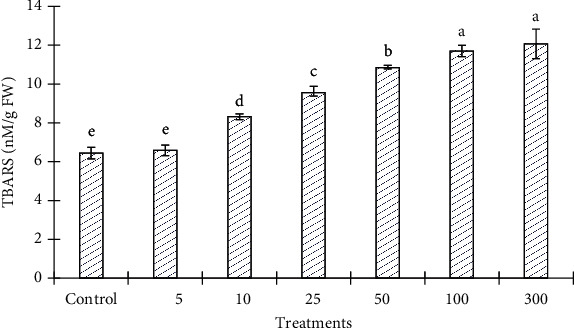
TBARS content in the leaves of *Ochradenus arabicus* treated with various concentrations of ZnONPs. Various letters on bars show the significant values according to Duncan's test (*p* < 0.05).

**Figure 10:**
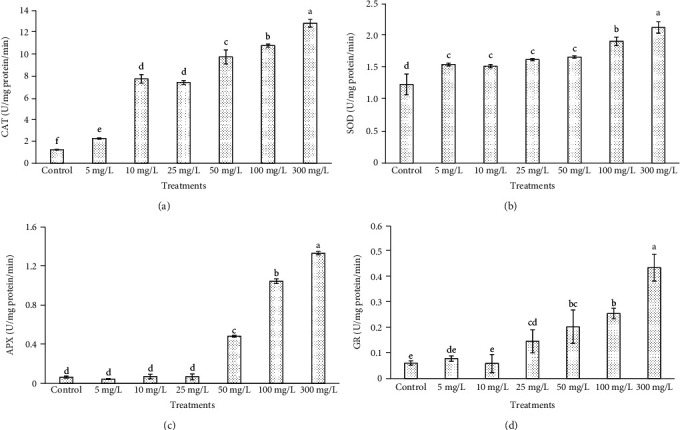
Antioxidant enzyme activities in leaves of *O. arabicus* with various concentrations of ZnONPs. Various letters on bars show the significant values according to Duncan's test (*p* < 0.05): (a) catalase; (b) superoxide dismutase; (c) ascorbate peroxidase; (d) glutathione reductase.

Chlorophyll content decreased at ZnONP concentrations, 25, 50, 100, and 300 mg/L, in a concentration-dependent manner (Figure 7). The level of chlorophyll content was increased with 5 and 10 mg/L of ZnONPs as compared to control and other shoot treatments. The levels of biochemical markers including proline and TBARS were found to be highest at 100 and 300 mg/L ZnONPs among the treatments and control (Figures 8 and 9). Both marker levels were increased as the concentration of ZnONPs increased in MS medium as compared to control. The antioxidant enzyme activities including CAT, SOD, APX, and GR were found to be higher with 300 mg/L ZnONPs than for the control (Figure 10) and other treatments. APX activity was found to be very low at low concentrations of ZnONPs (5, 10, and 25 mg/L), showing no significant difference compared with the control.

## 4. Discussion

The application of an optimal nanoparticle concentration is important to improve the growth and development of plants, as negative and/or positive effects might be observed at various concentrations. The optimal concentration of NPs induces growth and increases biomass in treated plants, as reported by many researchers. The impact of NPs also depends on the plant species, seed size, growth medium, growth stage, and material used for coating of nanoparticles [32]. Nanoparticles have been found to induce pronounced variations in the physiological indices in plants, such as percentage germination, root biomass, leaf number, and elongation [33]. Nanoparticles affect many factors, such as seed germination, biomass production, root growth, shoot growth, and biochemical and physiological activities [34].

In the current study, different concentrations of phytomediated ZnONPs were applied on regenerated shoots of *O. arabicus* cultured on MS medium. All culture conditions and the size of the explants were kept the same for the ZnONP-treated and control shoots. Cytokinin (BA) was used at a concentration of 0.1 mg/L on MS medium with ZnONPs, instead of BA (1 mg/L) used for shoot multiplication without NPs. Fresh weight and dry weight at 5 and 10 mg/L ZnONPs and shoot length at 10 and 25 mg/L ZnONPs were found to be higher significantly than that in the control (Figures 4 and 5). Our results are supported by a previous study [35] in which a low concentration of ZnONPs (1.5 ppm) produced the maximal promoting effect on shoot dry weight in *Cicer eritenum*. Our results are also supported by Zafar et al. [36], who found that 10 mg/L ZnONPs proved very effective for shoot emergence on MS medium in *Brassica nigra*. The application of 10 mg/L ZnONPs was also shown to induce callus and shoot regeneration in rapeseed [37] and plantlet growth in *Linum usitatissimum* [38]. The shoot proliferation rate in date palm was also found to be higher from buds cultured with ZnONPs on MS medium [39]. Moreover, the application of ZnONPs at 10 ppm on roots was shown to help to enhance the growth and yield of tomato plants [40]. ZnONPs promoted the shoot and root lengths and biomass in *Vigna radiata* [41].

A low concentration of nanoparticles improves growth and development in plants *in vivo* as well as *in vitro*, as reported above. The number of shoots was significantly increased in *O. arabicus* compared with control with ZnONP concentrations 10, 25, 50, 100, and 300 mg/L. The highest number of shoots was recorded with 50 mg/L ZnONPs, which was significantly different compared with all treatments (Figure 6). However, shoot length decreased in a concentration-dependent manner at 50–300 mg/L ZnONPs. Similarly, a high concentration of metal oxide nanoparticles caused phytotoxicity in plants, as reported by other researchers [42–44]. As the concentration of ZnONPs increased in MS medium, the biomass accumulation decreased, which might have been due to the production of reactive oxygen species. Our results are supported by the work of Zafar et al. [36], who showed that high concentrations of ZnONPs (500 to 1500 mg/L) adversely affected *Brassica nigra* seedling growth and seed germination and also led to increases in nonenzymatic antioxidants and antioxidative activities. The suspensions of zinc oxide nanoparticles at concentrations of 200–1000 mg/L affected seed germination in *Triticum aestivum* and decreased the level of photosynthetic pigments [45].

The photosynthetic rate depends on the chlorophyll content in the chloroplast, which plays an important role in plant growth and development. The chlorophyll content increased in shoots of *O. arabicus* treated with 5 and 10 mg/L ZnONPs compared with the level in the control; thereafter, it significantly decreased in a concentration-dependent manner (Figure 7). Thus, enhanced chlorophyll content in *O. arabicus* leaves at a low concentration of ZnONPs (5 and 10 mg/L) might play an important role in various growth parameters, such as shoot length and biomass production. Zinc deficiency was also reported to decrease chlorophyll synthesis in *Zea mays* L. [46]. Zinc plays important roles in many structural components of proteins and enzymes as a cofactor for pigment biosynthesis [47]. For example, in *Arabidopsis thaliana* (L.) Heynh, seedling growth, chlorophyll content, and rate of photosynthesis were reduced upon exposure to a high concentration of ZnONPs [48]. Moreover, ZnONPs were shown to inhibit the expression of genes involved in chlorophyll synthesis and photosystem structure [47]. An exposure of *Solanum melongena* to different concentrations of nanoparticles, namely, NiO, CuO, and ZnO, decreased its chlorophyll content [49].

Proline is an osmolyte that protects plant cells from biotic and abiotic stresses. It stabilizes subcellular structures in the cell and scavenges free radicals under various stress conditions [50]. The level of proline in *O. arabicus* changed with increasing ZnONP concentration on MS medium, compared with that in the control. The highest proline content was found to be 746.74 *μ*g/g FW in shoots treated with 100 mg/L ZnONPs, which was significantly higher than that in the other treatments as well as control (Figure 8). Helaly et al. [51] performed an experiment on banana regeneration in which proline content increased in the callus and shoots upon exposure to nanozinc and ZnONPs. Similarly, proline was increased in tomato plants upon exposure to ZnONPs at 8 mg/L, compared with the level in control plants [52]. Moreover, foliar treatment with various concentrations of nano-ZnO or ZnO to flax plants gradually increased proline to a level significantly higher than that in the control [53, 54]. Saradhi and Mohanty [54] reported that proline protects cells against free radical- and singlet oxygen-induced damage resulting from excess reactive oxygen species (ROS).

Thiobarbituric acid reactive substances (TBARS) are produced as byproducts of lipid peroxidation, resulting from oxidative stress, and can be measured by the TBARS assay using thiobarbituric acid as a reagent [22]. The TBARS content increased in all shoots treated with ZnONPs compared with the level in the control, in a concentration-dependent manner. The highest TBARS (12.063 nM/g FW) accumulation was found to occur with 300 mg/L ZnONPs, which was higher than in the other treatments and the control (Figure 9). However, no significant difference was found in TBARS accumulation in shoots of *O. arabicus* upon the application of 5 mg/L ZnONPs compared with the control. Our results are in line with the findings of Kumari et al. [55], who performed an experiment on *Allium cepa* with ZnONPs (nonbiogenic) and noted the accumulation of TBARS in a concentration-dependent manner.

Antioxidant defense systems play potential roles in plant protection under various stresses. The scavenging of ROS is necessary to avoid potential oxidative damage, for which various mechanisms play important roles in the cell. Enzymatic and/or nonenzymatic mechanisms have been shown to be involved in the antioxidant defense systems of plants [56, 57]. The activity of antioxidant enzymes was shown to be increased in *in vitro* shoot culture of *Arabidopsis thaliana* [38] upon the application of NPs. Moreover, the enzyme activities of CAT and SOD significantly increased in shoots of *O. arabicus* compared with those in the control at all concentrations of ZnONPs. The changes in activity of CAT and SOD were observed to be nonsignificant between the treatments at 10 and 25 mg/L ZnONPs; however, they remained significantly higher than that in the control. The activity of APX was observed to be very low in the shoots treated with 5, 10, and 25 mg/L ZnONPs, showing no significant differences compared with the control. In the same way, changes in GR activity were found to be very low upon treatment with 5 and 10 mg/L ZnONPs, showing no significant differences compared with the control. This might have been due to the involvement of ZnONPs in various metabolic activities of the cell, and application of low concentration of it played an important role in cell protection from ROS and induced growth and biomass accumulation. Several studies have reported that ZnONPs induced positive effects on the growth and development of crop plant species. ZnONPs have been widely used because zinc is an essential micronutrient and participates in various metabolic reactions [58]. Similarly, a low concentration of ZnONPs reduced the adverse effects generated by Cd in *Lycopersicon esculentum* and increased the activities of nitrate reductase and carbonic anhydrase, as well as protein content, compared with the levels in the control in both unstressed and stressed plants [40]. It has also been reported that the toxicity of ZnONPs on crop plants is much lower than that of bulk ZnO or Zn^2+^ particles [59].

The CAT, SOD, APX, and GR activities increased in a concentration-dependent manner with 50, 100, and 300 mg/L ZnONPs. The highest enzyme activities were observed in the shoots treated with 300 mg/L ZnONPs, with CAT, SOD, APX, and GR showing activities of 12.857, 2.132, 1.332, and 0.436 U/mg/min protein, respectively (Figure 10). The increased levels of enzymes at a high concentration of ZnONPs may counteract oxidative stress [60]. SOD plays an important role in this as it catalyzes the dismutation of superoxide anion to H_2_O_2_ [61], and its increased activity may protect plants against oxidative damage [62, 63]. Our results are supported by the work of Zaeem et al. [38], who showed that superoxide dismutase activities were higher in shoots of *Linum usitatissimum* treated with 500 mg/L ZnONPs. Our results are also supported by Faizan et al. [52], who found that the activities of POX, CAT, and SOD increased upon the application of ZnONPs (at 8 mg/L) at 45 days after sowing as compared with the levels in control plants. Application of ZnO-NPs to As-stressed soybean plants resulted in the increase of SOD, catalase (CAT), ascorbate peroxidase (APX), and glutathione reductase (GR) and improved the growth of the plants [64]. Similarly, foliar spray of ZnO-NPs mitigates the negative impact of salt stress as the level of dismutase (SOD) and catalase (CAT) was increased and improved the growth of the *Lycopersicon esculentum* Mill. [65]. ZnONPs protect the photosynthetic apparatus in plants by enhancing the activities of antioxidative enzymes as well as increasing biochemical markers. ZnONPs added to MS medium induced SOD, CAT, and POX activity in banana and enhanced its tolerance to biotic stress [51].

## 5. Conclusion

Zinc oxide nanoparticles have become one of the most important metal oxide NPs in biological applications due to their numerous benefits. In the present study, synthesized phytomediated nanoparticles from *O. arabicus* and their application on the shoot of *O. arabicus* at a low concentration proved to be very effective for promoting the proliferation of multiple shoots and the production of high biomass. Moreover, low concentrations of ZnONPs with reduced cytokinin concentration promoted shoot proliferation in *O. arabicus*. However, high concentrations of ZnONPs caused toxicity, as revealed by analyses of biochemical and morphological markers which might be associated with greater production of ROS. Low concentrations of ZnONPs on *in vitro* raised shoots of *O. arabicus* could be used to boost the production of various secondary metabolites as plants have substantial medicinal value to cure various diseases. Thus, phytomediated synthesized nanoparticles are environmentally safe and could be used *in vivo* as well as *in vitro* for crops as well as medicinal plants for the improvement of their yield and quality traits.

## Data Availability

Data is available on request.

## Abbreviations

TBARS: Thiobarbituric acid reactive substances
CAT: Catalase
SOD: Superoxide dismutase
APX: Ascorbate peroxidase
GR: Glutathione peroxidase
MS: Murashige and Skoog
BA: 6-Benzylaminopurine
FTIR: Fourier transform infrared
TEM: Transmission electron micrograph
UV: Ultraviolet
ZnO: Zinc oxide
EU: Enzyme unit
EDTA: Ethylenediaminetetraacetic acid
PVP: *Polyvinylpyrrolidone*
NBT: *Nitro blue tetrazolium chloride*
DDW: Double-distilled water
TCA: Trichloroacetic acid
DMSO: *Dimethyl sulfoxide*
NADPH: Nicotinamide adenine dinucleotide hydrogen phosphate
GSSG: Glutathione disulfide.
